# Hyperspectral Image Classification with Capsule Network Using Limited Training Samples

**DOI:** 10.3390/s18093153

**Published:** 2018-09-18

**Authors:** Fei Deng, Shengliang Pu, Xuehong Chen, Yusheng Shi, Ting Yuan, Shengyan Pu

**Affiliations:** 1 School of Geodesy and Geomatics, Wuhan University, Wuhan 430079, China; fdeng@sgg.whu.edu.cn (F.D.); shengliangpu@163.com (S.P.); ztyuan@whu.edu.cn (T.Y.); 2 State Key Laboratory of Earth Surface Processes and Resource Ecology, Beijing Normal University, Beijing 100875, China; cxh1216@gmail.com; 3 State Environmental Protection Key Laboratory of Satellites Remote Sensing, Institute of Remote Sensing and Digital Earth, Chinese Academy of Sciences, Beijing 100101, China; shiys@radi.ac.cn; 4 State Key Laboratory of Geohazard Prevention and Geoenvironment Protection, Chengdu University of Technology, Chengdu 610059, China

**Keywords:** capsule network, hyperspectral, image classification, deep learning, possibility density

## Abstract

Deep learning techniques have boosted the performance of hyperspectral image (HSI) classification. In particular, convolutional neural networks (CNNs) have shown superior performance to that of the conventional machine learning algorithms. Recently, a novel type of neural networks called capsule networks (CapsNets) was presented to improve the most advanced CNNs. In this paper, we present a modified two-layer CapsNet with limited training samples for HSI classification, which is inspired by the comparability and simplicity of the shallower deep learning models. The presented CapsNet is trained using two real HSI datasets, i.e., the PaviaU (PU) and SalinasA datasets, representing complex and simple datasets, respectively, and which are used to investigate the robustness or representation of every model or classifier. In addition, a comparable paradigm of network architecture design has been proposed for the comparison of CNN and CapsNet. Experiments demonstrate that CapsNet shows better accuracy and convergence behavior for the complex data than the state-of-the-art CNN. For CapsNet using the PU dataset, the Kappa coefficient, overall accuracy, and average accuracy are 0.9456, 95.90%, and 96.27%, respectively, compared to the corresponding values yielded by CNN of 0.9345, 95.11%, and 95.63%. Moreover, we observed that CapsNet has much higher confidence for the predicted probabilities. Subsequently, this finding was analyzed and discussed with probability maps and uncertainty analysis. In terms of the existing literature, CapsNet provides promising results and explicit merits in comparison with CNN and two baseline classifiers, i.e., random forests (RFs) and support vector machines (SVMs).

## 1. Introduction

Hyperspectral imaging plays an important role in the accurate measurement, analysis, and interpretation of land scene spectra [1,2,3]. Hyperspectral image (HSI) [4] often consists of hundreds of spectral bands which provide the valuable information for many applications, i.e., land cover classification [5], target detection [6], precision agriculture [7], and water resource management [8]. Image classification is the most important technique for labeling categories of each pixel based on spatial-spectral information [9,10,11]. HSI classification has become a very active area of research in recent years [4,9]. Over the past decades, various classification methods, such as random forests (RFs) [12,13] and support vector machines (SVMs) [14,15], have been employed in HSI classification task [16,17]. However, most of these methods suffer from various challenges, such as the curse of dimensionality and the spatial variability of spectral signature, or they focus only on spectral variability.

Recently, deep learning techniques, such as stacked autoencoders (SAEs) [18] and convolutional neural networks (CNNs) [19], have shown considerable potential for high-order data classification [20] such as 3-D HSI classification [21]. The strong capacity for learning effective representations with multiple levels of abstraction for hyperspectral data allows these techniques to provide a special perspective for feature learning and reasoning [22,23,24]. The recent literature has shown that CNNs deliver state-of-the-art performance for HSI classification [25,26,27,28]. The most important advantage of CNNs is that the distributed features can be extracted with the weight-shared convolutional kernels through processing, i.e., convolution, spatial pooling, and full-connection, at different levels [11]. Hu et al. [21] proposed a five-layer CNN for 3-D HSI data using spectral information without considering the spatial context [29], and achieved a better classification performance than several traditional methods. They found that CNN could be used effectively for HSI classification after building an appropriate network architecture. However, the abovementioned frameworks only consider spectral signature, while the information regarding spatial domain is neglected [11].

Although CNNs have achieved state-of-the-art performance, there are two conspicuous flaws [30,31]: (1) their failure to consider spatial hierarchies between features; and (2) their lack of rotational invariance. Thus, Sabour et al. [30] presented a novel type of neural networks called capsule networks (CapsNets). Via the dynamic routing process (i.e., improving or replacing backpropagation) and vectoring the result (i.e., substituting for the scalar output), CapsNets may overcome the abovementioned shortcomings. Luo et al. [11] first tried to apply CapsNet to adapt 3-D HSI data. They compared a CNN-based approach with the CapsNet-based model and concluded that the CapsNet-based method did not provide the expected benefits, and no deep insight was revealed, e.g., whether the two networks are comparable. In general, a comparison of methods depends on the comparison of classification maps or accuracies. We tried to make CNN and CapsNet as comparable as possible to facilitate a more profound exploration or improvement, which was the purpose behind wrapping them together and designing a comparable paradigm of network architecture. Thus, the objectives of this work were to introduce the state-of-the-art CapsNet into HSI classification task and conduct a fair comparison between CapsNet and CNN.

**Figure 1:**
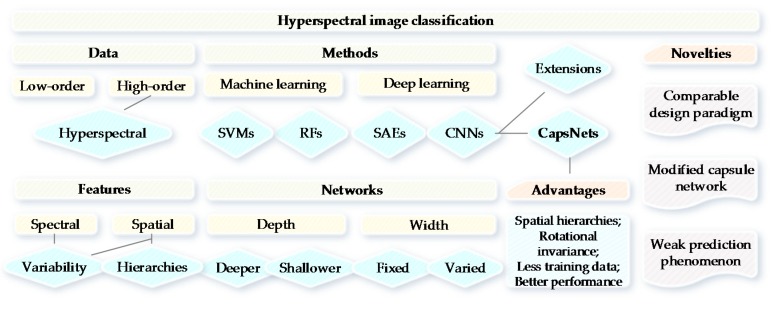
Overview of HSI classification with CapsNet.

The existing research for HSI classification often concentrates the information in the spectral and spatial domains (e.g., 3-D CNNs proposed in [29,32]), and considers the spectral and spatial variabilities; however, the spatial hierarchies between features have seldom been explored. Spatial hierarchies may consist of features, sizes, places, contexts, pyramids, or even ultrametrics based on the spatially perceptive relationships. The merits of our paper are that: (1) a modified two-layer CapsNet with limited training samples has been presented for HSI classification; (2) a comparable paradigm of network architecture design has been proposed for the comparison of CNN and CapsNet; and (3) CapsNet has much higher confidence for the predicted probabilities, which has been confirmed by probability maps and uncertainty analysis. CNNs have shown remarkable performance for HSI classification tasks, however, which involve a substantial amount of training samples [31]. The reduction of training data may be a favorable change for deep learning models. Makantasis et al. [33] presented a tensor-based scheme for HSI classification and analysis instead of CNN in the case when few training samples are available. In order to better clarify the intrinsic logic of this study, we provided a conceptual overview of HSI classification with CapsNet (see Figure 1).

The rest of this paper is structured as follows. The necessary background is introduced in Section 2, and the proposed CapsNet and a comparable paradigm of network architecture design are illustrated in Section 3. Experiments and analysis for the two HSI datasets are presented and discussed in Section 4 and Section 5, respectively. Finally, the conclusions of this work are summarized in Section 6.

## 2. Related Work

In this section, we provide a brief introduction to CNNs and CapsNets.

### 2.1. CNNs

**Figure 2:**
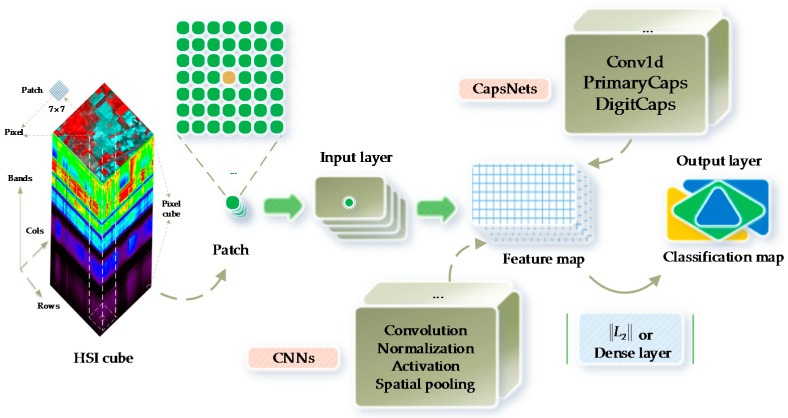
A typical CNN which is composed of an input layer, an output layer, the convolution, normalization, spatial pooling, and fully connected layers. A typical CapsNet consists of an input layer; an output layer; the Conv1d, PrimaryCaps, and DigitCaps layers; a 3-D hyperspectral cube with 200 bands; and a 3-D input patch with size 7 × 7. The 3-D HSI patches are preprocessed for deep learning models. The pixel cubes are fed into the conventional classifiers, i.e., RFs and SVMs.

CNNs are a type of deep neural network architectures based on shared weights (i.e., parameter sharing) [24]. Additionally, CNNs have been the most popular framework in recent years. The existing literature has shown that CNNs present better performance for HSI classification than conventional machine learning algorithms [19,26]. A typical CNN (see Figure 2) is composed of an input layer and an output layer as well as many hidden layers that are alternately stacked convolution, normalization, spatial pooling (i.e., downsampling or subsampling), and fully connected (i.e., dense) layers [21,24,27]. The convolutional layer extracts feature maps by the kernel-size-specified linear convolutional filters followed by the nonlinear activation functions (e.g., rectified linear units (ReLU)). The normalization layers often normalize input data to zero mean and unit variance, and then apply a linear transformation. The spatial pooling layers group the local features together from spatially adjacent pixels to improve the robustness of neural network to the slight deformations of objects. After several convolutional layers and pooling layers, the fully connected layers can perform high-level reasoning such as classifiers.

### 2.2. CapsNets

CapsNets (or CAPs) represent a completely novel type of deep learning architectures that attempt to overcome the limits and drawbacks of CNNs [30], such as lacking the explicit notion of an entity and losing valuable information during max-pooling. A typical CapsNet (see Figure 2) is shallower, with three layers, i.e., the Conv1d, PrimaryCaps, and DigitCaps layers. Sabour et al. [30] presented a capsule-based representation of a group of hidden neurons, in which not only likelihood but also the properties of the hidden features could be captured. In such a scenario, CapsNet was robust to affine transformation and required fewer training data. In addition, CapsNet has resulted in some special breakthroughs related to spatial hierarchies between features. A capsule represents a group of neurons [30]. The activities of neurons enclosed in an active capsule represent the various properties of a particular entity. In addition, the overall length of a capsule represents the existence of an entity, and the orientations of a capsule indicate its properties. A CapsNet is a discriminatively trained multi-layer capsule system [30]. Because the length of the output vector represents the probability of existence, an output capsule is computed using a nonlinear squashing function:

(1) $$v_{j}=\frac{{\left\|s_{j}\right\|}^{2}}{\varepsilon +{\left\|s_{j}\right\|}^{2}}\frac{s_{j}}{\left\|s_{j}\right\|},$$

where $v_{j}$ is the vector output of the capsule j and $s_{j}$ is its total input. The nonlinear squashing function is an activation function to ensure that the short vectors get shrunk to almost zero length and the long vectors get shrunk to a specific length with respect to ε.

(2) $$s_{j}=\sum_{i}c_{ij}W_{ij}u_{i}.$$

The total input to a capsule $s_{j}$ is obtained by multiplying the output $u_{i}$ of a capsule by a weight matrix $W_{ij}$, which represents a weighted sum over all predicted vectors from the capsules in the layer below. Here, $c_{ij}$ denotes a coupling coefficient that is determined by the iterative dynamic routing process:

(3) $$c_{ij}=\frac{\exp\left(b_{ij}\right)}{\sum_{k}\exp\left(b_{ik}\right)},$$

where $b_{ij}$ and $b_{ik}$ are the log prior probabilities between two coupled capsules.

In every capsule layer, each capsule outputs a local grid of vectors to each type of capsule in the layer above, and then the different transformation matrices for each grid member and each capsule type are used to obtain an equal number of classes. The dynamic routing [30] is implemented under an iterative process. A lower-level capsule sends its output to a higher-level capsule whose vectors have a large scalar product with the prediction coming from the lower-level capsule. The overall length of the output vector represents the predicted probability [30]. A separate margin loss *L_k_* for each capsule *k* can be given as

(4) $$L_{k}=T_{k}\max{\left(0,m^{+}-\left\|v_{k}\right\|\right)}^{2}+\lambda \left(1-T_{k}\right)\max{\left(0,\left\|v_{k}\right\|-m^{-}\right)}^{2},$$

where *T_k_* = 1, *m*^+^ = 0.9, and *m*^−^ = 0.1 are three free parameters by default. *λ* enables down-weighting of the loss and helps ensure final convergence.

Because CapsNets are recently proposed neural network architectures, only a few studies have explored the applications. Xi et al. [31] attempted to find the best set of configurations that yielded the optimal test error on complex data. Afshar et al. [34] adopted and incorporated a CapsNet for brain tumor classification and proved that it could overcome the defects of CNNs successfully. Jaiswal et al. [35] presented a generative adversarial capsule network which was a framework that used a CapsNet instead of the standard CNN as discriminators. Kumar et al. [36] proposed a novel method for traffic sign detection using a CapsNet that achieved outstanding performance. LaLonde and Bagci [37] expanded the use of CapsNets to the task of object segmentation for the first time and achieved a promising segmentation accuracy. Li et al. [38] built a CapsNet to recognize rice composites from UAV images. Qiao et al. [39] captured the high-level features using a CapsNet to reconstruct image stimuli from human fMRI, achieving higher accuracy than all existing state-of-the-art methods. Wang et al. [40] proposed a CapsNet structure based on a recurrent neural network for sentiment analysis. Zhao et al. [41] were the first to investigate empirically CapsNets for text modeling. Therefore, CapsNets have the potential abilities in most research fields and deserve attention. Concerning HSI classification, Luo et al. [11] first modified CapsNet, and attempted to apply it to adapt 3-D HSI data. However, they did not find the benefits of CapsNet in comparison with CNN. Actually, CapsNets are still in their infancy [42]. Hence, there have been few documented studies on the applications at present.

## 3. Proposed Approach

In this section, we describe the details of the proposed approach. When making a fair comparison between deep learning models, it is very important to keep their structures, sizes, and settings as similar as possible. The design principles of network architecture and CapsNet with parameter settings are described in the following subsections.

### 3.1. Network Design

**Figure 3:**
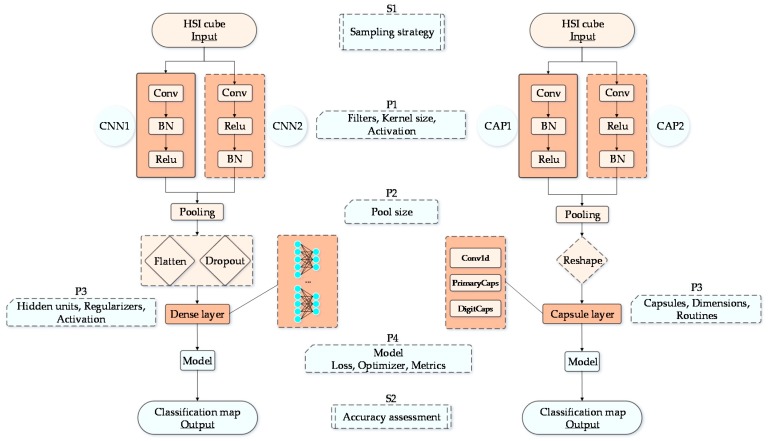
Proposed CNN and CapsNet. Here, Si {i = 1, 2} denotes the key sub-flows, and Pj {j = 1, 2, 3, 4} represents a set of parameter settings. CNN1 paired with CNN2 and CAP1 paired with CAP2 are the intertwined relationships with few differences. Note that CNN and CapsNet are completely independent networks herein.

The proposed paradigm of network architecture design is illustrated in Figure 3. The main objective of the designed network architecture is to create a comparable paradigm to suppress any diversity, however small, and to have the comparable aspects between CNN and CapsNet. Moreover, we tried to improve CapsNet to reap the expected benefits compared to CNNs. Given that neural networks with deeper layers may not always have better classification results for HSI classification [43], both CNN and CapsNet are relatively smaller, shallower neural networks. As shown in Figure 3, there is only one convolutional layer (i.e., a feature extraction layer) in the network architecture, and all models are two-layer network structures. The differences between CNN1 (or CAP1) and CNN2 (or CAP2) are the former contains a composite block “conv-bn-relu”, whereas the latter one incorporates an equal number of the stacked layers with batch normalization (BN) layer and ReLU layer reversed. First, the input data (i.e., 3-D patches) are sent to the convolutional layer, and the data size is (P_Row_, P_Col_, B_ands_), where B_ands_ is the number of channels for a 3-D hyperspectral cube. The kernels are of size 4 × 4, where the number of filters is 64, and the stride is taken as 1 by default. Then, the convolutional layer (i.e., Conv) is followed by a normalization layer.

As mentioned before, CNNs can extract features in the lower layer correctly. Therefore, we hold only one of the convolutional layers for our network architecture. The followed BN layer is expected to speed up the subsequent training and reduce the sensitivity to network initialization. The pooling operation is a very primitive form of routing [30]. Its good performance and surprising effectiveness have made spatial pooling the chosen processing. The composite capsule layer actually is regards as the same functionality as a dense layer, which is expected to decode the final output into a specific mapping with the identified classes. Here, the number of capsules and the hidden units of the dense layer are equal to the number of classes. The dimension of the capsules is set to 64, and there are three routings. The final output of network is a (1, classes) vector. If the *i*th element (i.e., the predicted probability) in the vector has the maximum value, then the *i*th label is the predicted label for the input sample. Specifically, CNN or CapsNet are both two-parameter-layer structures nominally. Meanwhile, the number of layers is the total number of the convolutional layers and dense layers (or capsule layers).

### 3.2. Parameter Learning

In our experiments, each pixel and its neighbors in a 7 × 7 patch are taken as a single sample. Thus, the data size of each sample is 7 × 7 × B_ands_. In general, the size of the convolutional filters in CNN or CapsNet is W, where W can be 3, 5 or higher. The 1 × 1 convolutional kernel has been excluded because it can extract features only from the different bands and not in the spatial domain. Additionally, only one convolutional layer is employed and followed by a max-pooling layer. Thus, we set the convolutional kernel size to 4 × 4. A dropout layer can improve the performance by mitigating the overfitting problem via discarding some co-adaptation of hidden units. Using the dropout operation, the network can learn more robust features and reduce the effect of noise. Therefore, there is one dropout layer in CNN, and its probability is set to 0.6. In the training and inference procedure, the training samples are initially randomly divided into a few size-specific batches. The batch size is set to 64 in our experiments. CNN and CapsNet are each trained using a first-order gradient-based optimization of stochastic objective functions, referred to as the Adam algorithm. For each epoch in 200 epochs, only one batch is sent to the sequential models for training at a time. The training procedure does not stop until it reaches the predetermined maximum number of iterations except in the case of an early stop option. In the test procedure, the test samples are also fed into the sequential models, and the final predicted labels can be obtained by finding the maximum value in the output vector.

### 3.3. Sampling Strategy

As done in previously published studies, the spectral values are taken as a vector format (i.e., 1-D form) input to CNN or CapsNet to conduct HSI classification [44]. However, using the 1-D form of hyperspectral data does not take full advantage of the ability of neural networks to extract spatial hierarchical characteristics [45,46]. Therefore, one single patch (i.e., the pixel’s neighborhood or a square patch around the central pixel) with all spectrum bands is labeled as a category’s sample for the subsequent training and testing. To make both network structures as comparable as possible, all designed ground truth samples are split at a fixed size. Because the small datasets are used for training with the proposed structures, only the limited samples are chosen for training. After the training set is determined by randomly selecting 60 per class, the validation set is extracted with the same size as the training set. The rest of the samples from the training and validation sets are taken as the test set. Parameter analysis of the impact of the training size demonstrates that the determined size of the training set is suitable in this study. Due to the small size of the training set, the performance may depend on the selected training samples, i.e., a training set with good representation is likely to provide a better performance (and vice versa). Therefore, the classification accuracies may vary according to the selected training samples [27].

## 4. Results and Analysis

In this section, we compare CapsNet with CNN using accuracy metrics, i.e., overall accuracy (OA), average accuracy (AA) and Kappa coefficient (Kappa or K). The experimental results and analysis are reported in the following subsections.

### 4.1. Dataset Description

**Figure 4:**
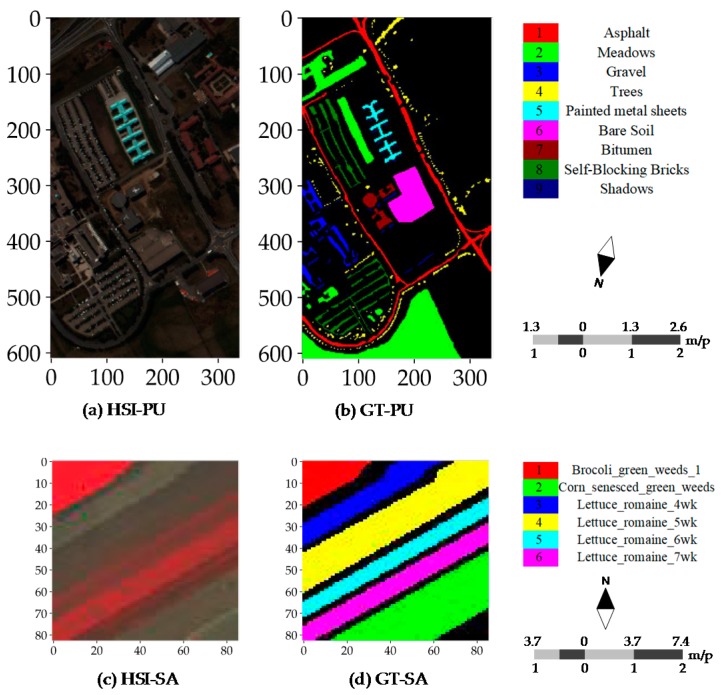
Real hyperspectral datasets: (**a**) pseudo-color image of the PU dataset; (**b**) ground truth samples of the PU dataset; (**c**) pseudo-color image of the SA dataset; and (**d**) ground truth samples of the SA dataset.

To evaluate CNN and CapsNet, two public real hyperspectral datasets (see Figure 4) with the different spatial resolution are used for the experiments. The PaviaU (PU) and SalinasA (SA) dataset are openly accessible online (http://www.ehu.eus/ccwintco/index.php?title=Hyperspectral_ Remote_Sensing_Scenes). The PU dataset (see Figure 4a,b) was collected over an urban area, and the SA dataset was collected in a natural area. The PU dataset was acquired by the ROSIS sensor during a flight campaign over Pavia University, northern Italy. The number of spectral bands is 103 after the thirteen noisiest bands were discarded. The hyperspectral dataset has a size of 610 × 340. Its spatial resolution is 1.3 m per pixel (m/p). There are nine classes included in the ground truth map and 42,776 labeled samples (Table 1). The PU dataset was provided by Prof. Paolo Gamba from Telecommunications and Remote Sensing Laboratory, the University of Pavia (Italy). The SA dataset (see Figure 4c,d) was acquired by the 224-band AVIRIS sensor over Salinas Valley, California, USA and is characterized by a high spatial resolution of 3.7 m per pixel. It comprises 83 × 86 pixels located within the Salinas scene. After the 20 water absorption bands were removed, 204 out of 224 bands were retained. The ground truth map is composed of 5348 pixels (see Table 1) and includes six land cover classes. As listed in Table 1, there are 42,776 labeled samples including nine categories in the PU dataset and 164,624 unlabeled samples are coded as class C0. There are 5348 labeled samples including six categories in the SA dataset and 1790 unlabeled samples are coded as class C0. The size of the training samples of all classes is set to 60 as same as the size of the validation samples. Except for the samples included in the training and validation sets, all other samples are taken for the test set. Apart from the importance of the selected training samples for determining the final outputs and accuracies, actually the type of dataset is another non-negligible factor. Moreover, the SA dataset has a large proportion of ground truth samples relative to the entire scene and the lower intra-class variability. These differences are crucial for investigating the robustness or representation of every model or classifier on the simple or complex data. This is why we used these two datasets.

### 4.2. Experimental Setup

Our experimental platform is a laptop equipped with an Intel Core i7-4810MQ 8-core 2.80 GHz processor, 16 GB of memory, and a 4G NVIDIA GeForce GTX 960M graphics card. The training procedures run on the GPU to achieve the high computational speeds. Because we train our networks with small training data, the training times of both datasets can be completed within a few minutes with the five independent runs and 200 epochs per run. It is rather fast and shows the high efficiency in terms of the shallower deep learning models.

For the proposed CapsNet, the additive convolutional layer has the convolutional kernels with a size of 4 × 4, 64 filters, a stride size of 1, and a “ReLU” activation. The later layers are a batch normalization layer and a max-pooling layer, which are included with the intention for the consistency with CNN. Note that the kernel size and filters should be adjusted to ensure that the capsule layer has the correct input dimension and acquires the qualified vectors. The final layer contains the 16-D capsules per class, and a capsule receives input from all capsules in the layer below. To ensure a complete comparison with CNN and to improve CapsNet, we kept the parameter settings of every model as similar as possible. We ran the experiments five times with every model or classifier for each dataset, and kept the sizes of the training and validation sets identical. These sets were randomly shuffled to reduce the influence of random effects, and finally the statistical accuracies were recorded.

### 4.3. Training Details

The training procedure for the PU dataset involves the determination of training samples and recording the accuracy and loss of training and validation procedures. The sampling strategy is critical to obtain a good performance for every model using the limited known samples. The accuracy and loss of training and validation procedure may depend on many factors, and show whether a model is qualified enough and its parameter configuration is ready for the subsequent parameter learning. Additionally, the spatial distribution of training and validation samples and the accuracy and loss of training and validation procedures with increasing iterations are plotted in the same subsection. The SA dataset covers a natural area, which has many ground truth samples relative to non-ground truth pixels, unlike the PU dataset. Moreover, the intra-class variability of every class in the SA dataset is relatively low. Consequently, all models and classifiers show a high performance.

**Figure 5:**
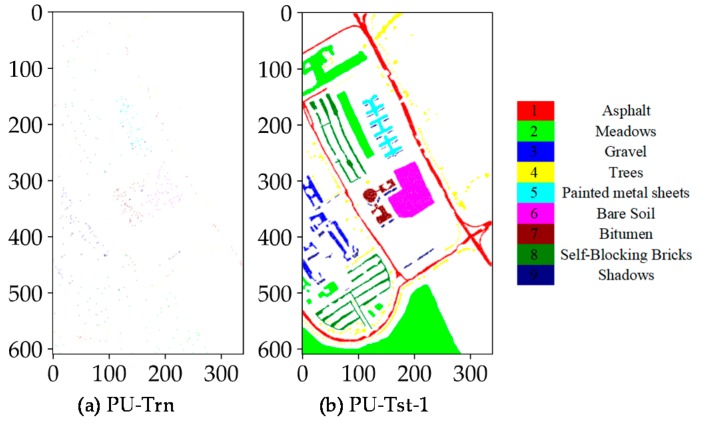

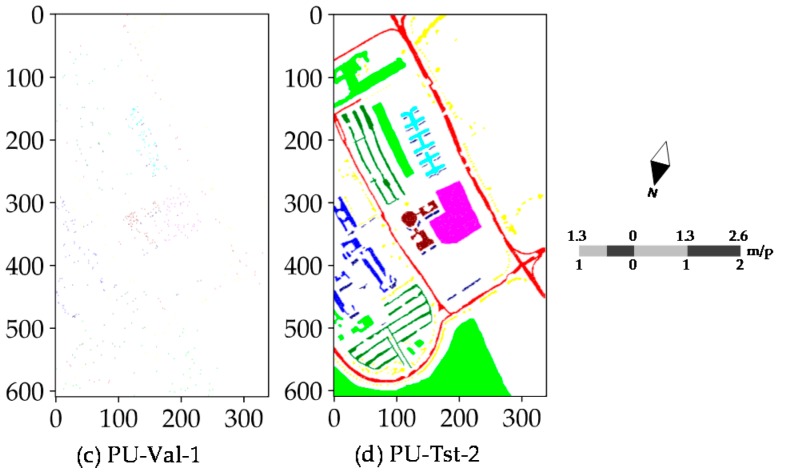
Spatial distribution of training, test and validation samples for the PU dataset: (**a**) training samples for fitting CNN and CAP, as well as RF and SVM classifiers; (**b**) test samples for predicting CNN and CAP; (**c**) validation samples for evaluating CNN and CAP; and (**d**) test samples for evaluating RF and SVM classifiers. “PU-Trn”, “PU-Tst-1” and “PU-Val-1” are the selected samples for training, testing and verifying CNN and CAP, respectively, while “PU-Trn” and “PU-Tst-2” represent the selected samples for training and testing RF and SVM classifiers, respectively.

**Figure 6:**
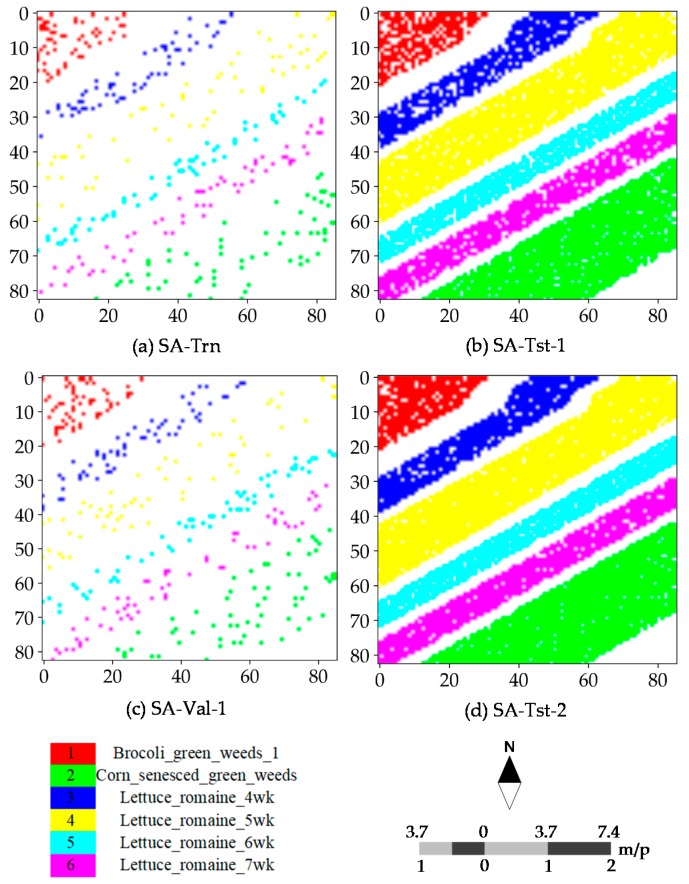
Spatial distribution of training, test and validation samples for the SA dataset: (**a**) training samples for fitting CNN and CAP, as well as RF and SVM classifiers; (**b**) test samples for predicting CNN and CAP; (**c**) validation samples for evaluating CNN and CAP; and (**d**) test samples for evaluating RF and SVM classifiers. “SA-Trn”, “SA-Tst-1” and “SA-Val-1” are the selected samples for training, testing and verifying CNN and CAP, respectively, while “SA-Trn” and “SA-Tst-2” represent the selected samples for training and testing RF and SVM classifiers, respectively.

Figure 5 and Figure 6 illustrate the training, test and validation sets (i.e., the central pixels) with randomly selected 60 samples per class for the PU and SA dataset. For nine classes (Class 1–Class 9), the scattered samples of the different classes have exactly the same color as the ground truth classes. These sparse samples are fed into the different models and classifiers to determine the best model weights and parameters for the subsequent inference and label assignment. The spatial distribution of the designed sets illustrated is the first one of five independent runs. For the four other independent runs, the training samples will be randomly selected at a fixed size as well. For each run, 60 samples per class are randomly selected for training and the additional 60 samples for validation, while the rest of samples are available for testing.

**Figure 7:**
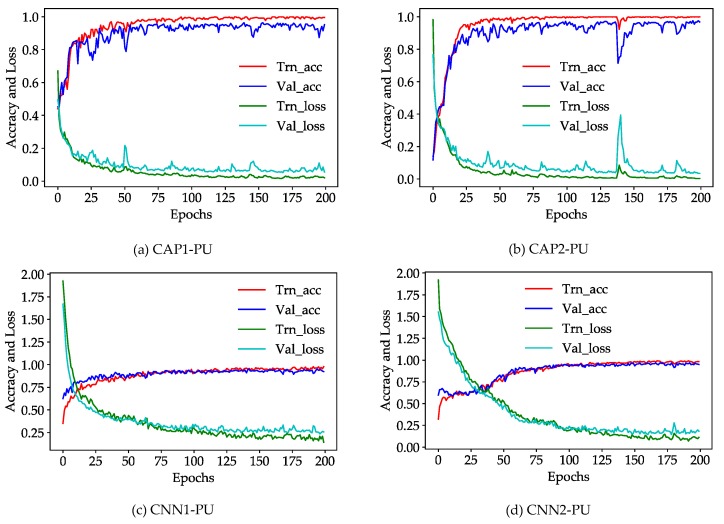
Accuracy and loss of: (**a**) CAP1; (**b**) CAP2; (**c**) CNN1; and (**d**) CNN2 for the PU dataset with increasing iterations where the horizontal axis represents 200 epochs per independent run.

According to Figure 7, for the PU dataset, the CNN- and CAP-based models appear to converge either locally or globally, and show the good convergence behavior. The CAP-based models stabilize much faster at ~50 epochs, and the CNN-based models attain a stability at ~100 epochs. In addition, the CNN-based models show a better representation of the local convergence than the CAP-based models. Additionally, all independent runs show good convergence. As for the SA dataset, all models have some difficulties in handling the local portions because the SA dataset is a relatively simple dataset. Therefore, some problems may arise, such as vanishing gradients for every independent run. Note that the unnecessary PCA transformation or layer normalization should be avoided in terms of a simple dataset considering the possible problems such as exploding gradients.

Fine-tuning the hyperparameters of classifiers allows us to obtain a set of optimal parameters. The parameters of RF and SVM classifiers can be optimized by the cross-validated grid search over a parameter grid to obtain a robust baseline classifier. In this study, we fine-tuned five parameters of RF classifier, i.e., the quality criterion of a split, the maximum depth of tree, the number of features, the minimum number of samples required to split, and the number of trees in the forest. Two parameters, i.e., the penalty parameter of the error term and the kernel coefficient for “rbf”, were fine-tuned for SVM classifier. The implementation of SVM classifier was based on “libsvm” with a one-vs.-one scheme. Moreover, all grid searches were calculated using the five-fold cross-validation.

For the PU and SA datasets with RF classifier, the best parameters obtained in the first independent run are as follows: (1) the Gini impurity, which is taken as the quality criterion; (2) the maximum depth, determined as 8, which is the value until all leaves contain less than the minimum number of samples; (3) the maximum number of features, which is calculated by a square root operation of the number of features; (4) the minimum number required to split an internal node, considered to be 2; and (5) the number of trees, which is optimized to 32. Finally, the best score is 0.7593 and 0.9639, respectively. For the PU and SA datasets with SVM classifier, the best parameters obtained in the first independent run are the penalty parameter, which is fixed at 10,000.0 and 1000.0, respectively, and the kernel coefficient for “rbf”, which is determined to be 0.01 and 0.1, respectively. Finally, the best score is 0.8074 and 0.9833, respectively.

### 4.4. Classification Maps

When the training procedure is successfully completed, the next step is to have the unlabeled samples categorized into the proper classes. In such a case, two kinds of scenes can be obtained. One is the whole scene covering the raw hyperspectral image, and another covers all ground truth samples referred to as the reference scene. Classification maps for the PU dataset have the distinct features owing to being an urban area, the wider coverage, and more categories.

**Figure 8:**
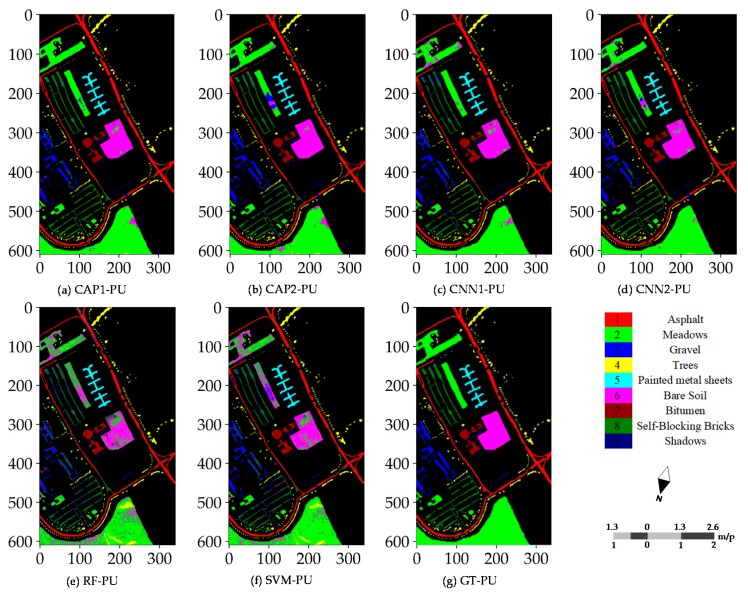
Classification maps (reference scene) of: (**a**) CAP1; (**b**) CAP2; (**c**) CNN1; (**d**) CNN2; (**e**) RF; and (**f**) SVM for the PU dataset; and (**g**) ground truth samples of the PU dataset.

**Figure 9:**
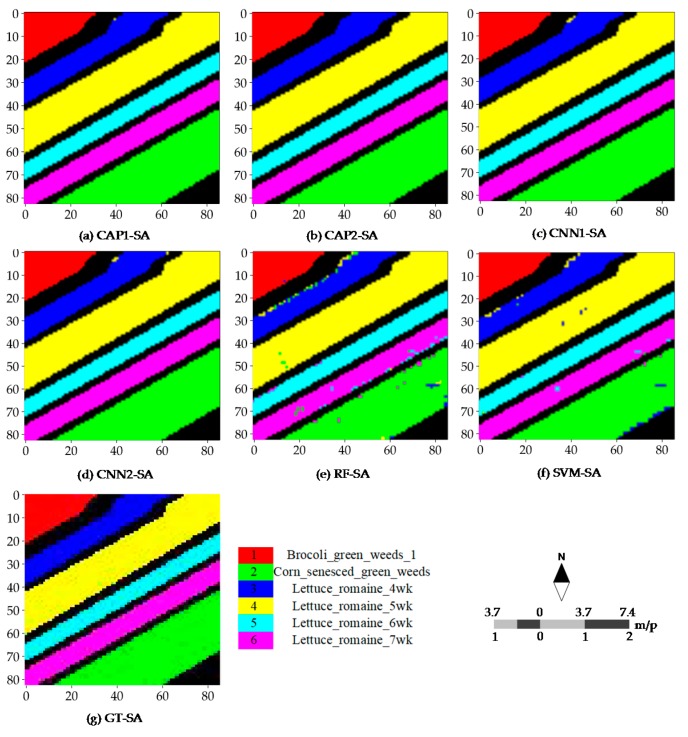
Classification maps (reference scene) of: (**a**) CAP1; (**b**) CAP2; (**c**) CNN1; (**d**) CNN2; (**e**) RF; and (**f**) SVM for the SA dataset; and (**g**) ground truth samples of the SA dataset.

Figure 8 illustrates the different classification maps obtained by the CNN- and CAP-based models for the PU dataset with 60 randomly selected training samples per class. Misclassifications are mainly caused by some fragmental structures or intra-class variability. The results of the subsequent accuracy assessment, and the probability maps also support such a conclusion. Probability maps are utilized to observe the probability density and find the weak predictions. The CNN- and CAP-based models have promising outputs compared to the two baseline classifiers, i.e., RF and SVM classifiers. Furthermore, most errors of commission and omission occur in the non-homogeneous areas involving the complex landscape structures or materials. Such results are commonly seen in the different random runs. Classification maps of every model or classifier for the SA dataset are rather satisfactory except the bottom-right corner and cross-area (i.e., the transitional buffer) spanning the different sampling regions. Good performance is observed in Figure 9. Misclassifications are mainly caused by some inherent uncertainties between classes. Because the SA dataset is a relatively simple dataset, all models obtain fairly the good results and accuracies, and surpassing the conventional baseline classifiers, i.e., RF and SVM classifiers.

### 4.5. Classification Accuracies

Several widely used accuracy metrics, i.e., the OA, AA, K, and accuracy of each class, are used to assess the final classification results. These metrics are derived from the site-specific confusion matrix. For the PU dataset, regarding CAP1, many samples of Class 1 (Asphalt) are wrongly classified as Class 7 (Bitumen), and several samples of Class 6 (Bare Soil) are classified as Class 2 (Meadows). Concerning CAP2, many samples of Class 1 are wrongly classified as Class 7, and many samples of Class 2 are classified as Class 6. The apparent omission errors appear in Class 1, and Class 8 (Self-Blocking Bricks) is meant to have a considerable intra-class variability. With respect to CNN1, the apparent errors occur between Class 1 and Class 7, Class 2 and Class 6, and Class 3 (Gravel) and Class 8. As for the two baseline classifiers, except for Class 5 (Painted metal sheets) and Class 9 (Shadows), the other classes have obvious omission errors and commission errors. For Class 4 (Trees) with RF and SVM classifiers, many samples are wrongly classified as Class 2, and more samples of Class 2 are incorrectly classified as Class 4. The apparent commission and omission errors between the class pairs demonstrate the low inter-class variability. The classification accuracies obtained for the SA dataset are fairly good. For all models and classifiers, there are no obvious commission and omission errors. Such results indicate the superior performance (i.e., the saturated accuracies) when using a relatively simple dataset.

Based on Table 2, the classification accuracies can be summarized as follows: (1) both network structures show the promising performance; (2) the CAP-based models are slightly superior to the CNN-based models in terms of the Kappa, OA, and AA; and (3) most of the commission and omission errors occur in Class 1 (Asphalt) and Class 7 (Bitumen), Class 2 (Meadows) and Class 6 (Bare Soil), and Class 3 (Gravel) and Class 8 (Self-Blocking Bricks). In addition, compared to classification accuracies of the CNN presented by Yu et al. [27], our CNN-based models get better performance. Table 3 shows that: (1) all models and classifiers get the nearly saturated performances; (2) most of the commission and omission errors are caused by Class 2 (Corn_senesced_green_weeds) and Class 3 (Lettuce_romaine_4wk); and (3) CAP and CNN have the similar accuracies for all classes.

### 4.6. Probability Maps

**Figure 10:**
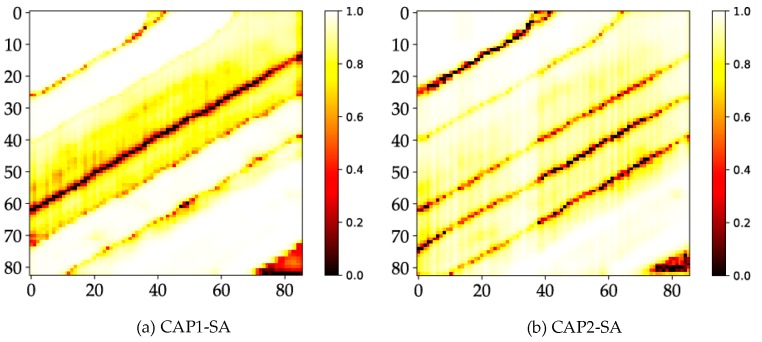

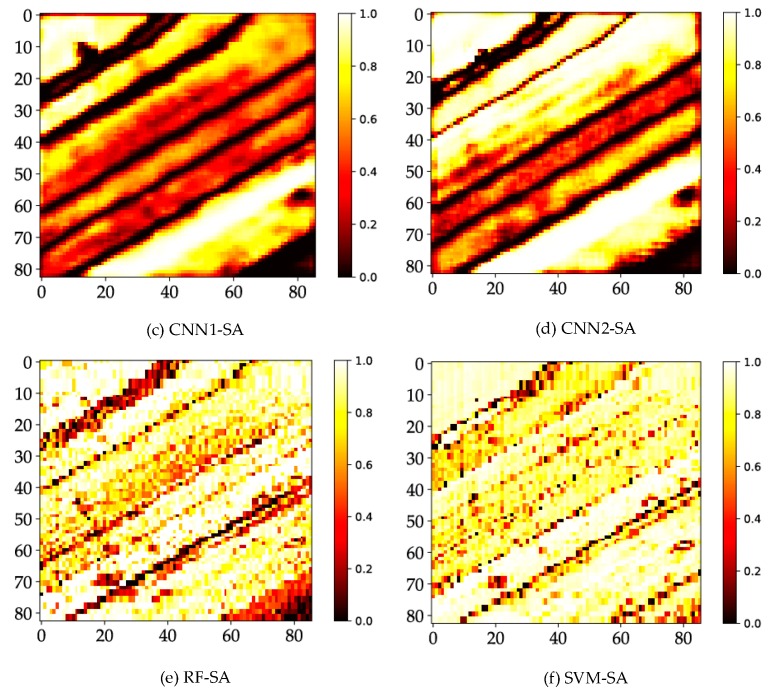
Probability maps of: (**a**) CAP1; (**b**) CAP2; (**c**) CNN1; (**d**) CNN2; (**e**) RF; and (**f**) SVM for the SA dataset.

Additionally, to show that CAP has clear advantages over CNN, we graph the probability maps of every model or classifier. An apparent distinction is reflected in the probability maps of CNN and CAP. This result discloses that the differences of the simple and complex datasets have a significant impact on the measuring performance. Figure 10 shows noticeable differences between the CNN- and CAP-based models, such as the weak predictions in a holistic scene in the case of the CNN-based models. Thus, the maximum predicted probabilities of many samples are relatively low. Weak predictions occur in the cross-area and the area covered by non-ground truth samples in the case of the CNN-based models. Furthermore, the CAP-based models for the SA dataset are supposed to be consistent with the results of the PU dataset.

## 5. Discussion

### 5.1. Parameters Determination

The performance of HSI classification with the supervised neural networks [43] usually depends on: (1) the representative ability of the designed structures; (2) the number of training samples; (3) the size of input patch: and (4) the parameter configurations (or the experimental setups). We take 7 × 7 as the patch size based on the information given in the literature [29,43,47]. The batch size (i.e., 64) of the input data is of particular importance to obtain a proper representation of the accuracy and loss of training and validation procedures. Regarding the influence of model sizes, deep learning models with the deeper layers appear to have worse classification results for HSI classification [43]. Meanwhile, shallower network structures are apt to be comparable to each other. Previous work has indicated that a 3 × 3 kernel size encounters with the serious overfitting problem, and the 1 × 1 convolutional kernel can provide better performance [27]. We found that a 4 × 4 kernel size and 64 filters for one convolutional layer are a viable option. Additionally, Zhong et al. [48] also found that deep learning models with the regularization methods (i.e., BN) could obtain the higher classification accuracy than those without. The spatial pooling operation has been retained due to its superior performance. Yu et al. [27] suggested that the dropout operation was necessary and provided a better performance. Additionally, for the capsule layer, the dimension of capsules is set to 64, and three routings are used by default.

Random runs (or Monte Carlo runs) are especially useful for obtaining credible results through the repeated random sampling. Hence, we applied every model and classifier to each dataset five times. We illustrate the first run and discuss the others below. For the five independent runs, 60 samples per class were randomly selected for training and an additional 60 samples for validation, while the remaining samples were available for testing. Every run shows a slight difference because there is an inherent randomness in neural networks. The accuracies are recorded to calculate the final statistical mean and standard error. Because the training samples for each run are randomly selected from the same category, these runs are expected to give nearly consistent results regardless of the possible noise or contamination. Different from repeating many times, the random runs use the different training sets every time, which can achieve the more reliable classification results at the risk of the possible decrease of the final accuracy statistics.

### 5.2. Impact of Training Size

Generally, if raw HSI data are formatted as an n-D input, then a tensor-based learning approach can be applied. The tensor-based learning [20] is a promising technique for the high-order data classification where a HSI cube is the typical 3-D high-order data. The exploitation of the high-order complex data raises the new research challenges due to the high dimensionality and the limited number of ground truth samples [49]. The number of training samples is a critical factor in determining the performance of a model or classifier. Deep learning models may not extract effective features unless abundant training samples are available [47]. For HSI data, a sufficient number of training samples may be difficult; hence, determining the appropriate number of training samples for HSI classification is critical. Zhong et al. [43] reported that most models obtained a better performance for the larger training sets. Therefore, we chose 60 randomly selected samples per class.

**Figure 11:**
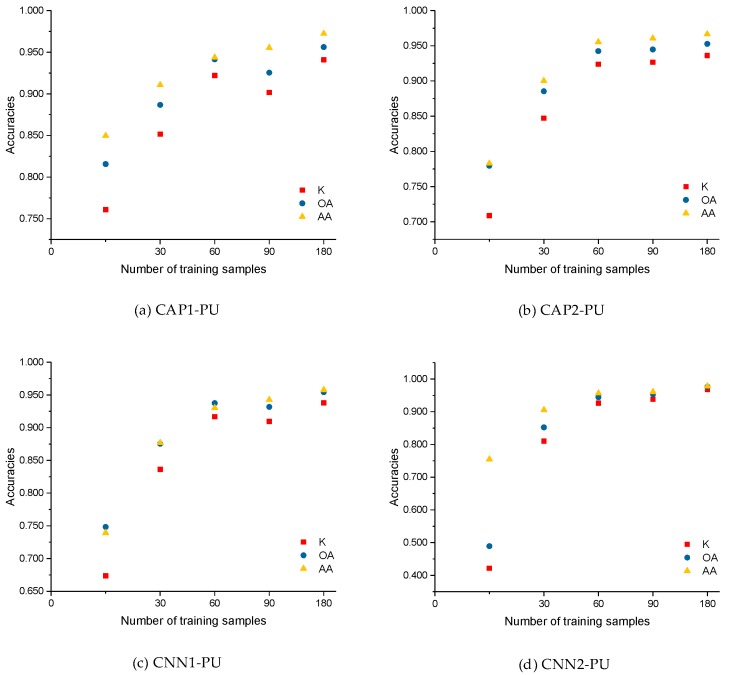

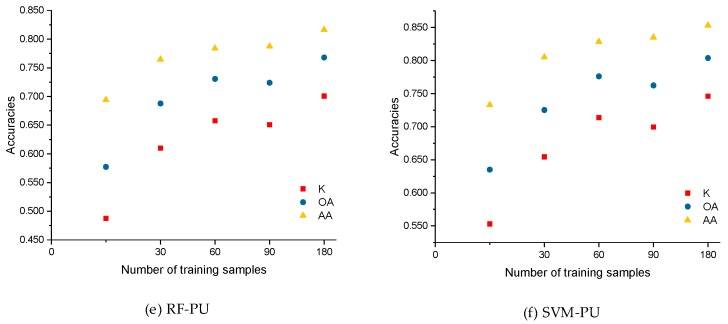
Accuracies of: (**a**) CAP1; (**b**) CAP2; (**c**) CNN1, and (**d**) CNN2; (**e**) RF; and (**f**) SVM for the different training sizes of the PU dataset.

As shown in Figure 11, we defined the number of the training samples to be 15, 30, 60, 90, and 180 (i.e., 0.25, 0.5, 1.5, and 3 times the base case). The optimal number of the training samples was determined to be 60 according to the experimental analysis. However, as the number of training samples increases, the classification accuracies are continuously improved and become somewhat stable. For CAP1 in the case of size 15, K, OA, and AA are 0.7609, 81.56%, and 84.96%, respectively, compared to the corresponding values yielded by CNN1 of 0.6735, 74.82%, and 73.93%. Similarly, for CAP2, K, OA, and AA are 0.7086, 77.95%, and 78.27%, respectively, while 0.4215, 48.90%, and 75.46% for CNN2. Here, the CAP-based models can achieve better performance with much fewer training data. Regarding the two baseline classifiers, i.e., RF and SVM classifiers, the classification accuracies increase smoothly as the number of the training samples increases. The parameter analysis of the impact of the training size supports the determination of the number of training samples in this study.

### 5.3. Uncertainty Analysis

Probability density is not necessarily consistent with the final reliability of the classification output. However, it is still expected to somehow indicate the confidence of the classification output, because the predicted probabilities are estimated based on the distribution of training samples. Actually, the output probabilities of a classifier or deep learning model have been considered as the uncertainty of classification result for the consequent analysis in many previous studies [50,51,52,53]. Therefore, the information of output probabilities is very helpful. Related work on uncertainty analysis, or the statistics of the predicted probabilities, have seldom been considered in HSI classification task before. This work provides us the new insight into the intrinsic differences between CNN and CAP, when putting the concerns of performance measure aside.

**Figure 12:**
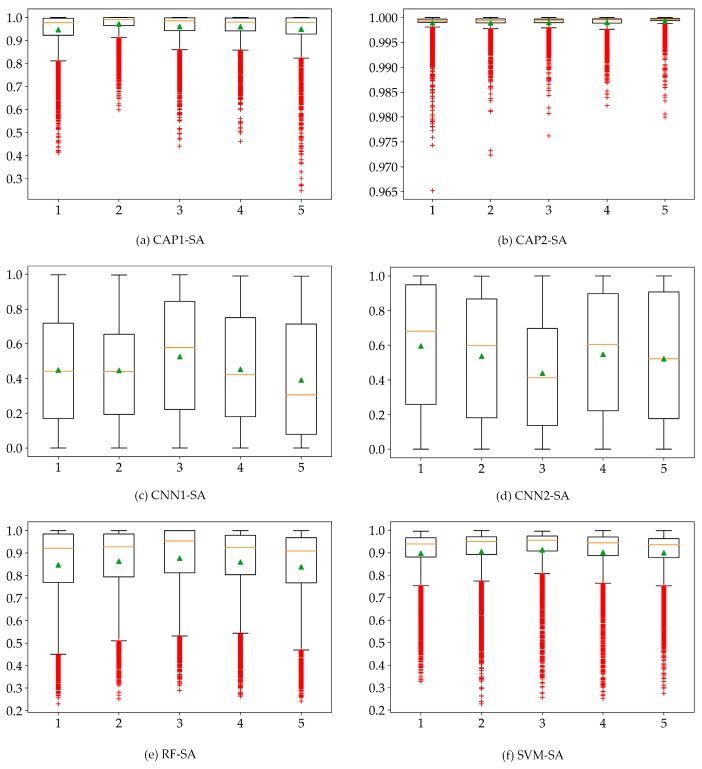
Box plots of the predicted probabilities for: (**a**) CAP1; (**b**) CAP2; (**c**) CNN1; and (**d**) CNN2 models; (**e**) RF; and (**f**) SVM using the SA dataset.

Label assignment relies on the credible predictions, and the maximum predicted probabilities would determine the final outputs. As shown in Figure 12, the CAP-based models show a high prediction confidence where most of the predicted maximum probabilities are fairly good. The performance of the CNN-based models may be sufficient in terms of the final accuracies. However, the predicted maximum probabilities of most samples are relatively low. For RF and SVM classifiers, their representations appear normal. We speculate that such variance may depend on the complexity of the experimental data. Based on the experimental results, both CNN and CAP produced classification results with the very high accuracy. However, the predicted probabilities of CNN are much smaller than the true reliability of its classification output. It is apparent that the presented CAP obtains a maximum probability for the category prediction and label assignment. Consequently, CAP shows much higher confidence for the predicted probabilities which has been analyzed by probability maps and uncertainty analysis (i.e., the statistical analysis). The probability map illustrates the observed spatial distribution (i.e., probability density) of the weak predictions, and the statistical analysis indicates the statistical distribution of the predicted probabilities. The weak predictions occur in the cross-area (i.e., the transitional buffer) and the areas covered by non-ground truth samples. Concerning the statistical plots, there are speculations can be given as follows: (1) if most predictions have a high probability, then the low probabilities will be outliers, and vice versa; (2) if most predictions are quite approximate, then no outliers can be identified; and (3) the floated box with its properties such as height and size may indicate a specific statistical distribution. The output probabilities may be intentionally manipulative considering the possible operations such as activation (or squashing) functions which can limit the output signal to a finite value. However, the statistical distribution is supposed to be consistent all the way. For the scope of this study, CAP shows the preferable and exceptional characteristics.

### 5.4. Time Consumption

The training time provides a direct measure of the computational efficiency [43], and which is also an important performance indicator for the different network structures. Network parameters play an important role in the computational complexity of deep learning models. Specifically, once the network parameters are fixed, the efficiency of every model can be determined approximately. In general, a designed network structure with a specific dataset can be used to determine the total network parameters. Accordingly, the changes in the magnitudes of network parameters would alter the training and test times. Differences in the network parameters between CNN and CAP depend primarily on the differences between the capsule layer and dense layer in terms of a comparable paradigm of network architecture design. Note that we attempt to make both network structures as comparable as possible, thus facilitating further improvement.

With respect to the time consumption (see Table 4), CAP costs more time than CNN. From another perspective, both network structures have approximately the same network complexity, as we expected. CAP requires approximately 1.8 times more time than CNN for the PU dataset. Furthermore, it can be speculated that, when the data complexity increases, more time would be taken by CAP and SVM. This means that the time consumption of CAP and SVM may depend on the data complexity. Furthermore, the conventional SVM classifier exhibits a promising efficiency with little time consumption, and RF classifier has the much larger computational intensity. Yu et al. [27] trained their networks with small training sets and the training was completed in several hours, while some other applications presented by Krizhevsky et al. [54] required days or weeks. We have tried our best to improve the efficiency of CNN and CAP in our study, i.e., run on the GPU, use small training data, make a proper division of training samples, design a shallower deep learning architecture (i.e., a two-parameter-layer structure), etc. Therefore, CNN or CAP can be trained within a minute. On the other hand, CNN and CAP have a different size of network parameters that would cause the inevitable but reasonable differences in time consumption. Additionally, the training time may depend on many possible factors, i.e., the randomness in neural networks, the influence of memory storage, and the difference of computational environment, etc.

## 6. Conclusions

CapsNets are a recently proposed type of deep learning network architectures, and only limited studies have explored the applications. Motivated by the innovativeness of CapsNets, we attempted to introduce CapsNets into HSI classification task. In this paper, we propose a modified two-layer CapsNet for HSI classification. Two real HSI datasets, the PaviaU and SalinasA datasets, are taken as the benchmark datasets. Compared with the state-of-the-art CNN and two baseline classifiers, i.e., RF and SVM classifiers, the proposed CapsNet achieves better classification results for the complex data, even with limited training samples. In addition, a comparable paradigm of network architecture design has been proposed to compare CNN and CapsNet. Additionally, we observed that CapsNet has much higher confidence for predictions. We observed weak predictions with regard to the probability density, which are confirmed by probability maps and uncertainty analysis. Since CNNs may fail to retain the spatial and spectral coherency of samples, the latest emerged approaches, such as the tensor-based learning algorithms and CapsNets, may be the appealing alternatives using less training data but a relatively good performance. To our knowledge, CNNs have several complex extensions including varied depth and width, with the composite functions and specific tricks on the operations of the input, hidden, and output layers. The complexity of CapsNets has not been well explored until now. Since further efforts should be devoted to improving CapsNets, the comparison between more complex CNNs and CapsNets may be an open field to be exploited hereafter. Meanwhile, classification maps and accuracies may always be qualified for evaluating a classifier or deep learning model. In light of this study, we believe that CapsNets have bright prospects in HSI classification, and further efforts should be devoted to improving CapsNets in the near future.

## Figures and Tables

**Table 1:** Definitions of ground truth classes and samples for the PU and SA datasets.

Dt	Cn	Class	Samples	Train	Test	Val
PU	C0	Non-ground truth	164,624	0	0	0
	C1	Asphalt	6631	60	6511	60
	C2	Meadows	18,649	60	18,529	60
	C3	Gravel	2099	60	1979	60
	C4	Trees	3064	60	2944	60
	C5	Painted metal sheets	1345	60	1225	60
	C6	Bare Soil	5029	60	4909	60
	C7	Bitumen	1330	60	1210	60
	C8	Self-Blocking Bricks	3682	60	3562	60
	C9	Shadows	947	60	827	60
SA	C0	Non-ground truth	1790	0	0	0
	C1	Brocoli_green_weeds_1	391	60	271	60
	C2	Corn_senesced_green_weeds	1343	60	1223	60
	C3	Lettuce_romaine_4wk	616	60	496	60
	C4	Lettuce_romaine_5wk	1525	60	1405	60
	C5	Lettuce_romaine_6wk	674	60	554	60
	C6	Lettuce_romaine_7wk	799	60	679	60

**Table 2:** Classification accuracies for the PU dataset with five runs.

	CAP1-PU	CAP2-PU	CNN1-PU	CNN2-PU	RF-PU	SVM-PU
K	0.9324 ± 0.0242	0.9456 ± 0.0181	0.9345 ± 0.0130	0.9332 ± 0.0221	0.6450 ± 0.0147	0.7070 ± 0.0094
OA	0.9490 ± 0.0185	0.9590 ± 0.0138	0.9511 ± 0.0097	0.9496 ± 0.0169	0.7189 ± 0.0124	0.7703 ± 0.0071
AA	0.9542 ± 0.0112	0.9627 ± 0.0138	0.9367 ± 0.0174	0.9563 ± 0.0089	0.7869 ± 0.0098	0.8273 ± 0.0096
C1	0.8943 ± 0.0545	0.9277 ± 0.0264	0.9468 ± 0.0279	0.9382 ± 0.0407	0.6604 ± 0.0143	0.7629 ± 0.0248
C2	0.9686 ± 0.0278	0.9659 ± 0.0210	0.9807 ± 0.0070	0.9507 ± 0.0272	0.6892 ± 0.0237	0.7374 ± 0.0082
C3	0.9229 ± 0.0312	0.8882 ± 0.1190	0.8518 ± 0.0782	0.8851 ± 0.0404	0.6364 ± 0.0284	0.7171 ± 0.0438
C4	0.9797 ± 0.0096	0.9761 ± 0.0121	0.9724 ± 0.0178	0.9720 ± 0.0124	0.9142 ± 0.0250	0.9042 ± 0.0259
C5	1.0000 ± 0.0000	1.0000 ± 0.0000	1.0000 ± 0.0000	1.0000 ± 0.0000	0.9928 ± 0.0052	0.9969 ± 0.0016
C6	0.9609 ± 0.0338	0.9781 ± 0.0284	0.9212 ± 0.0241	0.9483 ± 0.0321	0.7191 ± 0.0334	0.7738 ± 0.0324
C7	0.9831 ± 0.0137	0.9800 ± 0.0085	0.8783 ± 0.1855	0.9620 ± 0.0125	0.8296 ± 0.0205	0.8537 ± 0.0288
C8	0.8796 ± 0.0806	0.9483 ± 0.0224	0.8799 ± 0.0649	0.9504 ± 0.0196	0.6612 ± 0.0332	0.7015 ± 0.0380
C9	0.9985 ± 0.0009	0.9998 ± 0.0005	0.9993 ± 0.0006	1.0000 ± 0.0000	0.9793 ± 0.0072	0.9986 ± 0.0013

**Table 3:** Classification accuracies for the SA dataset with five runs.

	CAP1-SA	CAP2-SA	CNN1-SA	CNN2-SA	RF-SA	SVM-SA
K	0.9992 ± 0.0007	0.9991 ± 0.0004	0.9994 ± 0.0004	0.9991 ± 0.0003	0.9647 ± 0.0073	0.9845 ± 0.0035
OA	0.9994 ± 0.0005	0.9993 ± 0.0003	0.9995 ± 0.0003	0.9993 ± 0.0002	0.9720 ± 0.0058	0.9877 ± 0.0028
AA	0.9995 ± 0.0002	0.9992 ± 0.0003	0.9993 ± 0.0004	0.9989 ± 0.0003	0.9704 ± 0.0051	0.9883 ± 0.0025
C1	1.0000 ± 0.0000	1.0000 ± 0.0000	1.0000 ± 0.0000	1.0000 ± 0.0000	0.9940 ± 0.0019	0.9952 ± 0.0024
C2	0.9990 ± 0.0020	0.9987 ± 0.0019	1.0000 ± 0.0000	0.9997 ± 0.0007	0.9574 ± 0.0170	0.9782 ± 0.0056
C3	0.9984 ± 0.0008	0.9968 ± 0.0027	0.9956 ± 0.0027	0.9940 ± 0.0022	0.9266 ± 0.0070	0.9763 ± 0.0082
C4	0.9994 ± 0.0008	1.0000 ± 0.0000	1.0000 ± 0.0000	1.0000 ± 0.0000	0.9974 ± 0.0008	0.9944 ± 0.0041
C5	1.0000 ± 0.0000	1.0000 ± 0.0000	1.0000 ± 0.0000	1.0000 ± 0.0000	0.9954 ± 0.0052	0.9971 ± 0.0030
C6	1.0000 ± 0.0000	1.0000 ± 0.0000	1.0000 ± 0.0000	1.0000 ± 0.0000	0.9518 ± 0.0199	0.9886 ± 0.0055

**Table 4:** Network parameters and time consumption (i.e., the average time of five runs).

Models	PU (610 × 340 × 103)		SA (83 × 86 × 204)	
	Total Parameters	Training Time (s)	Total Parameters	Training Time (s)
CAP1	1.42 × 10^5^	35	2.33 × 10^5^	27
CAP2		37		27
CNN1	1.08 × 10^5^	21	2.10 × 10^5^	20
CNN2		21		20
RF	-	51	-	52
SVM	-	13	-	5
